# Supplementary material for: An intact C-terminal end of albumin is required for its long half-life in humans
Source: Commun Biol. 2020 Apr 20;3:181. doi: 10.1038/s42003-020-0903-7 (PMC7171077; doi:10.1038/s42003-020-0903-7)
Supplement: Supplementary file 1 — Supplementary Information [file 42003_2020_903_MOESM1_ESM.docx]

**Supplementary Figures**

**Supplementary Figure 1. Genetic HSA variants with a modified C-terminal end.** Recombinant WT HSA, Rugby Park, Venezia, Catania and L585X were expressed in HEK293E cells, purified using a human albumin affinity matrix and analyzed by SDS-PAGE and Coomassie staining.

**
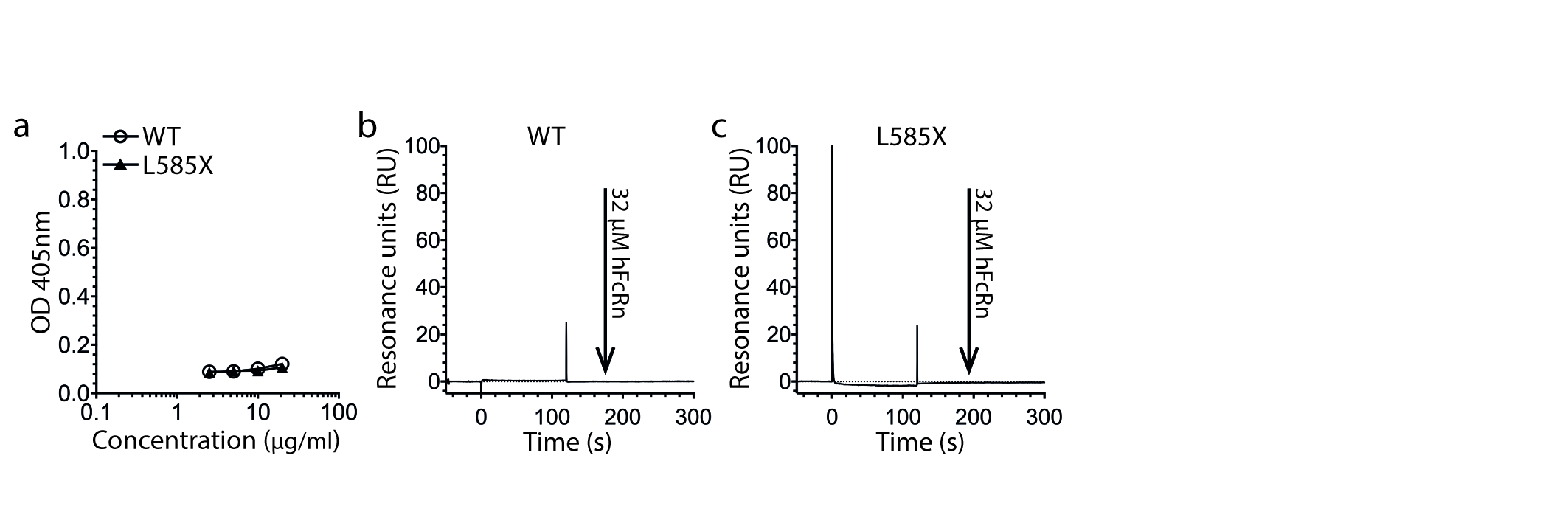
**

**Supplementary Figure 2. Binding of WT HSA and L585X to hFcRn at pH 7.4.** (**a**) ELISA responses of WT HSA and L585X binding to hFcRn at pH 7.4. (**b-c**) SPR responses obtained when 32 μM of monomeric hFcRn was injected over immobilized (~200-300 RU) (**b**) WT HSA and (**c**) L585X at pH 7.4. Injections were performed at 25°C and the flow rate was 40 ul/min.

**Supplementary Figure 3. HDX-MS analysis of WT HSA and L585X.** Deuterium uptake plots for 77 peptides (indicated) of WT HSA (black) and L585X (red). The “maximally labelled” control sample is shown as a data point in blue. The data points represent the mean ± s.d. of triplicates except for 2960 min, where n=1.

**
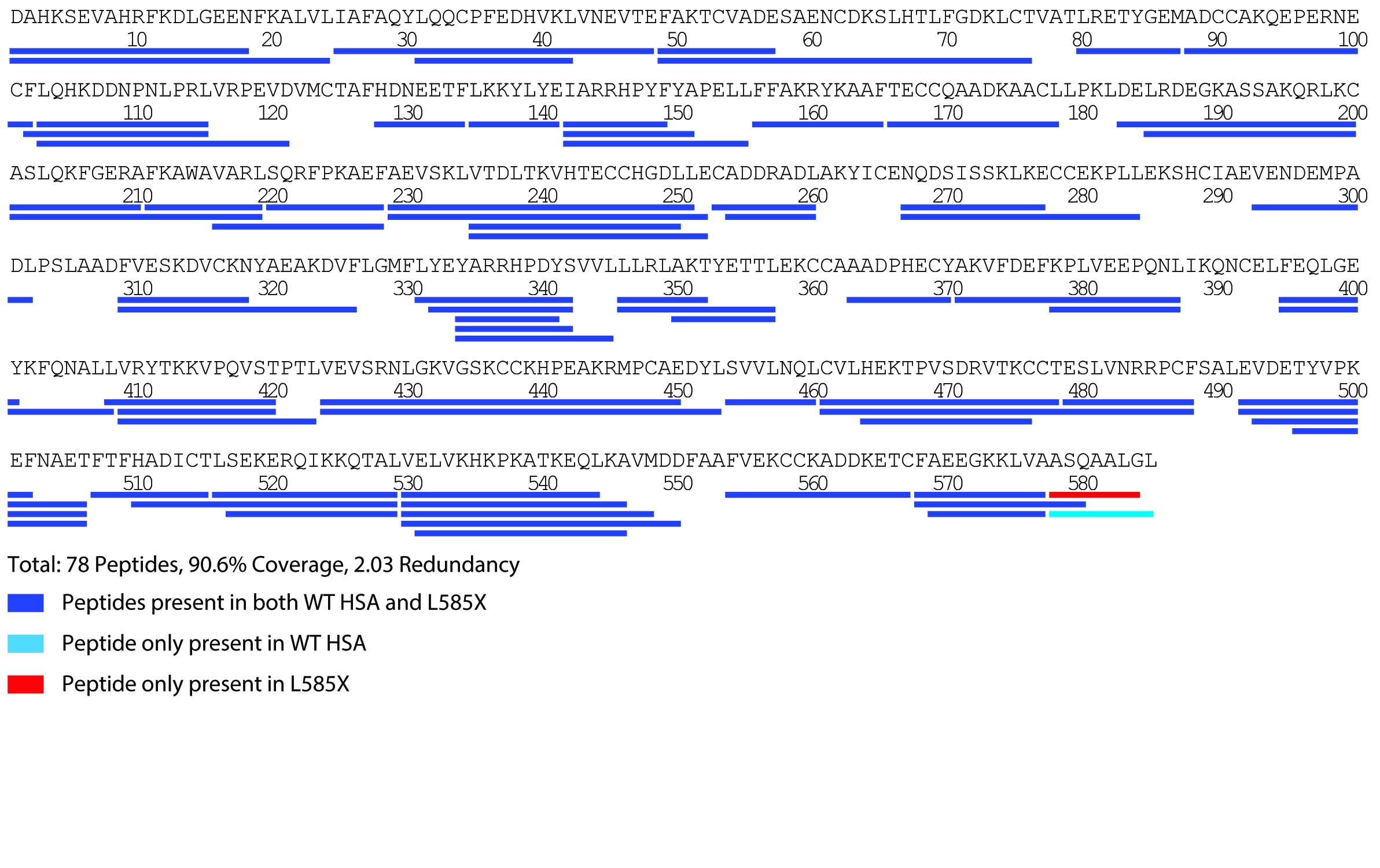
**

**Supplementary Figure 4. Effective sequence coverage map.** The blue bars corresponds to peptides for which useful HDX data were obtained. In areas without peptides, no structural information could be obtained. Reliable HDX data were obtained for 77 peptides covering 91% of the HSA sequence.


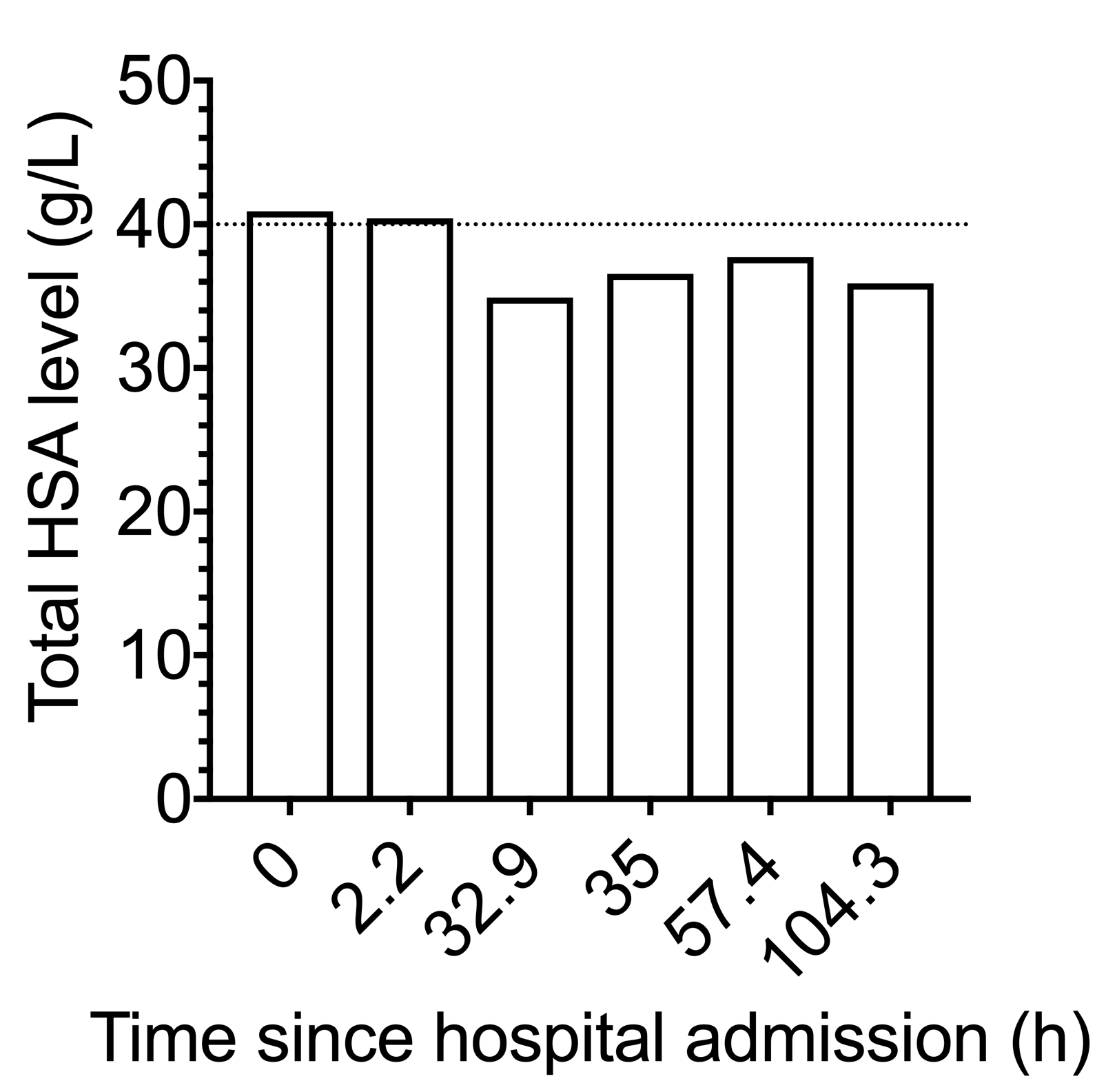


a

b

c

**Mass (Da)**

3% L585X of total HSA

66414

**Supplementary Figure 5. ESI-MS analysis of albumin in human plasma.** (**a-b**) The total concentration (**a**) and deconvoluted ESI-MS spectra (**b**) of HSA in blood collected over time from a patient with acute pancreatitis. The ESI-MS spectra shows two main peaks with the molecular weight indicated on the top, representing full-length HSA (black) and L585X (red). (**c**) Deconvoluted ESI-MS spectrum of HSA in plasma collected from a healthy control, which shows one main peak representing full-length HSA (66.4 kDa) and a signal of 3% at -113 Da representing L585X. The percentage of L585X of the total HSA is indicated.

**
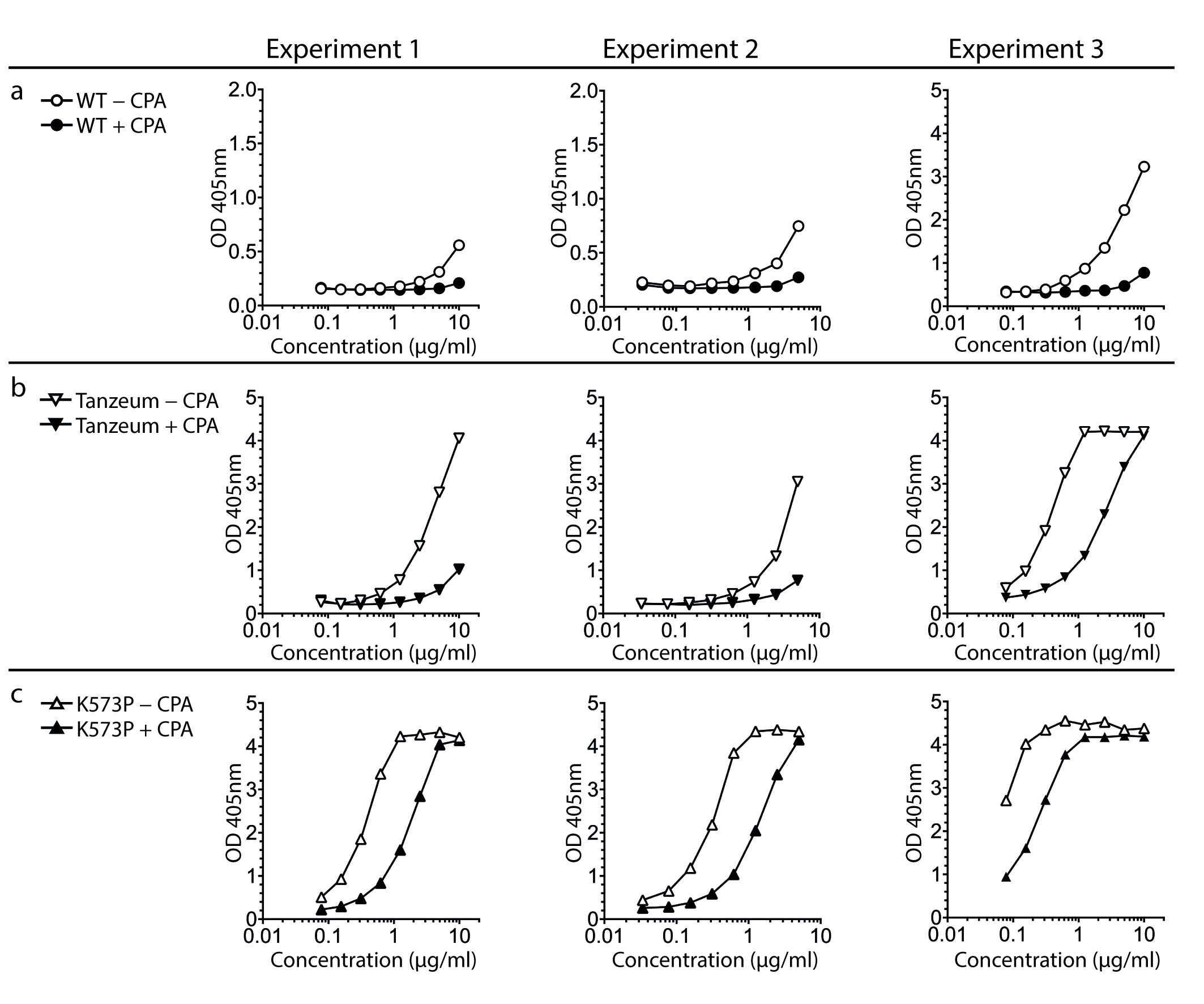
**

**Supplementary Figure 6. HSA variants show reduced hFcRn binding after exposure to CPA.** ELISA showing hFcRn binding at pH 5.5 of (**a**) WT HSA (circle), (**b**) Tanzeum® (downward triangle) and (**c**) K573P (upward triangle) exposed (filled black) or not exposed (filled white) to CPA.


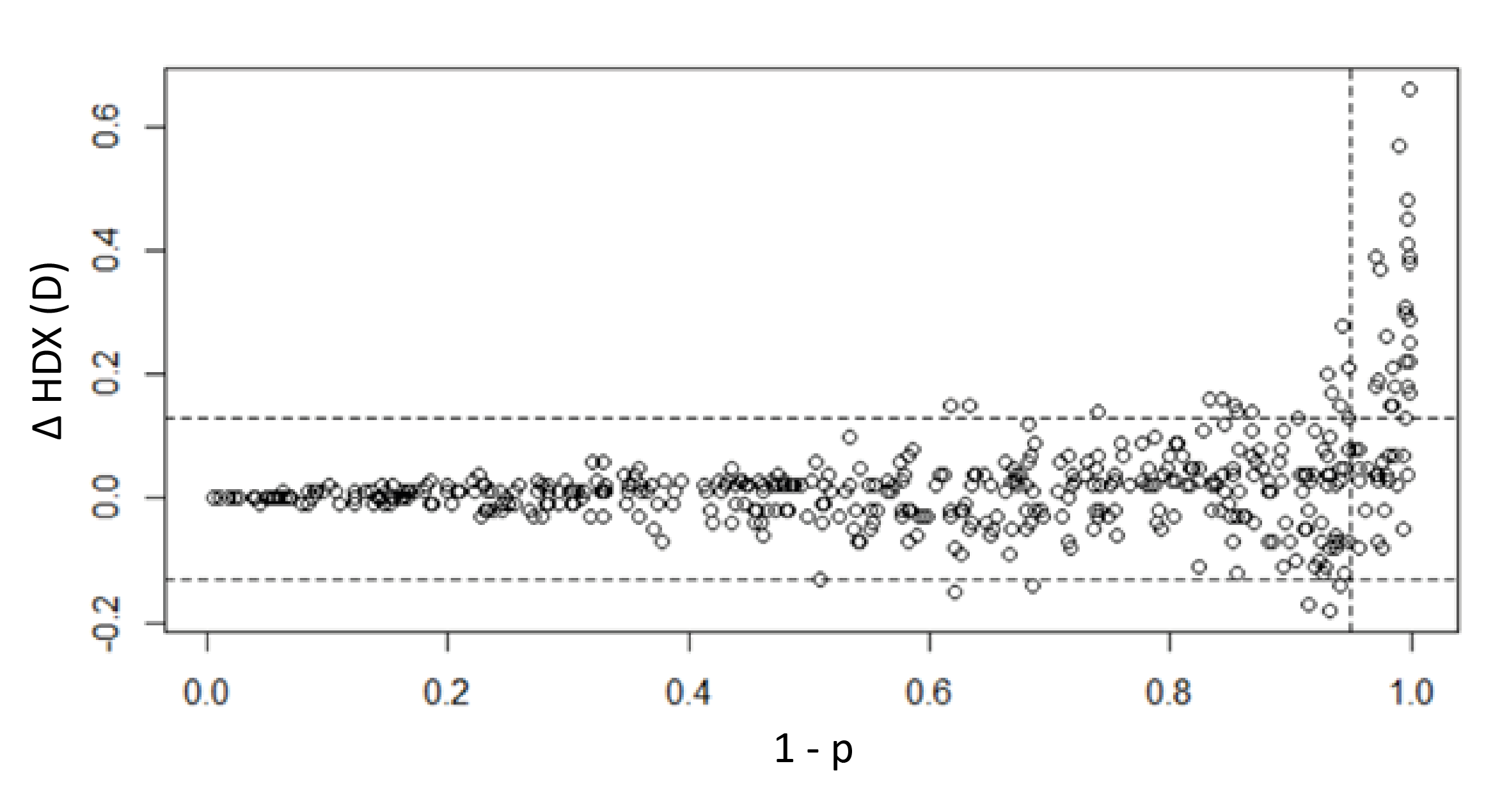


**Supplementary Figure 7. Scatterplot of replicate HDX data of the comparison of WT HSA and L585X.** The difference in HDX is plotted on the y-axis, while 1 subtracted the p-value of the result of a student’s t-test is plotted on the x-axis. Each point represents a single peptide at a single time point. The horizontal dashed lines represents a two times the pooled standard deviation *(± 2 ×* $\sqrt{{SD}_{wt}^{2}+{SD}_{L585X}^{2}}$ *= ± 0.13D*) cutoff based on the noise in the HDX measurements. The vertical dashed line represents a 1-p cutoff value of 0.95.

**Supplementary Tables**

**Supplementary Table 1. C-terminal peptides of HSA variants exposed or non-exposed to CPA**

| **C-terminal peptides** | **HSA WT**  **- CPA** | **HSA WT**  **+ CPA** | **Tanzeum**  **- CPA** | **Tanzeum**  **+ CPA** | **HSA KP**  **- CPA** | **HSA KP**  **+ CPA** |
| --- | --- | --- | --- | --- | --- | --- |
| LVAASQAALG- | 5,04x10^8^ | 1,95x10^10^ | 9,82x10^7^ | 1,53x10^10^ | 1,90x10^8^ | 9,67x10^9^ |
| KLVAASQAALG- | 2,42x10^7^ | 6,23x10^9^ | 5,47x10^6^ | 6,29x10^9^ | n.d. | 6,52x10^6^ |
| LVAASQAALGL | 3,90x10^10^ | 4,56x10^7^ | 4,04x10^10^ | 1,34x10^8^ | 2,47x10^10^ | 6,35x10^6^ |
| KLVAASQAALGL | 4,45x10^9^ | n.d. | 7,94x10^9^ | n.d. | n.d. | n.d. |
| ADDKETCFAEEGPKLVAASQAALGL | 1,83x10^7^ | n.d. | n.d. | n.d. | 6,79x10^9^ | n.d. |
| ETCFAEEGPKLVAASQAALGL | n.d.^a^ | n.d | n.d. | n.d. | 6,32x10^6^ | n.d. |
| **Percentage lacking L585** | **1,21** | **99,82** | **0,21** | **99,93** | **0,60** | **99,93** |

^a,^ n.d., not detected

**Supplementary Table 2. HDX data summery**

| **Data Set** | **WT** | **L585X (mut)** |
| --- | --- | --- |
| HDX reaction details | 111.2 mM Na_2_HPO_4_, 44 mM citric acid, pD_read_ = 5.530, 25 °C | 111.2 mM Na_2_HPO_4_, 44 mM citric acid, pD_read_ = 5.530, 25 °C |
| HDX time course (min) | 0.25, 1, 10, 60, 480, 1440 | 0.25, 1, 10, 60, 480, 1440 |
| HDX control samples | Maximally-labeled control  (Protein level) | Maximally-labeled control  (Protein level) |
| # of Peptides | 77 | 77 |
| Sequence coverage | 91 % | 91 % |
| Average peptide length / Redundancy | 13.86 / 2.03 | 13.84 / 2.03 |
| Replicates (biological or technical) | 3 (labelling) | 3 (labelling) |
| Repeatability (Average standard deviation / D) | 0,046 | 0,0457 |
| Significant difference (delta HDX > X D) | 0.246 D | |
| Average back-exchange (% / IQR) | 42.70 / 13.9 | |
