# Supplementary material for: An intact C-terminal end of albumin is required for its long half-life in humans
Source: Commun Biol. 2020 Apr 20;3:181. doi: 10.1038/s42003-020-0903-7 (PMC7171077; doi:10.1038/s42003-020-0903-7)
Supplement: Supplementary file 2 — Description of Additional Supplementary Files [file 42003_2020_903_MOESM2_ESM.pdf]

Description of Additional Supplementary Files

Supplementary Data 1: HDX data underlying graphs presented in Fig. 3 and Supplementary Figure 3.

Supplementary Data 2: Source data underlying graphs presented in Fig. 1, Fig. 2, Fig. 4 and Fig. 5.
