# Supplementary material for: An intact C-terminal end of albumin is required for its long half-life in humans
Source: Commun Biol. 2020 Apr 20;3:181. doi: 10.1038/s42003-020-0903-7 (PMC7171077; doi:10.1038/s42003-020-0903-7)
Supplement: Supplementary file 5 — Reporting Summary [file 42003_2020_903_MOESM5_ESM.pdf]

nature research

Corresponding author(s): Jan Terje Andersen

Last updated by author(s): Mar 4, 2020

Reporting Summary

Nature Research wishes to improve the reproducibility of the work that we publish. This form provides structure for consistency and transparency in reporting. For further information on Nature Research policies, see Authors & Referees and the Editorial Policy Checklist.

Statistics

For all statistical analyses, confirm that the following items are present in the figure legend, table legend, main text, or Methods section.

n/a	Confirmed	
☐	☒	The exact sample size (*n*) for each experimental group/condition, given as a discrete number and unit of measurement
☐	☒	A statement on whether measurements were taken from distinct samples or whether the same sample was measured repeatedly
☐	☒	The statistical test(s) used AND whether they are one- or two-sided<br>*Only common tests should be described solely by name; describe more complex techniques in the Methods section.*
☒	☐	A description of all covariates tested
☐	☒	A description of any assumptions or corrections, such as tests of normality and adjustment for multiple comparisons
☐	☒	A full description of the statistical parameters including central tendency (e.g. means) or other basic estimates (e.g. regression coefficient) AND variation (e.g. standard deviation) or associated estimates of uncertainty (e.g. confidence intervals)
☐	☒	For null hypothesis testing, the test statistic (e.g. *F*, *t*, *r*) with confidence intervals, effect sizes, degrees of freedom and *P* value noted<br>*Give P values as exact values whenever suitable.*
☒	☐	For Bayesian analysis, information on the choice of priors and Markov chain Monte Carlo settings
☒	☐	For hierarchical and complex designs, identification of the appropriate level for tests and full reporting of outcomes
☒	☐	Estimates of effect sizes (e.g. Cohen's *d*, Pearson's *r*), indicating how they were calculated

Our web collection on statistics for biologists contains articles on many of the points above.

Software and code

Policy information about availability of computer code

Data collection	Biacore 3000 Control software 4.1 and Biacore T200 Control software 2.0.1 were used to collect the SPR data; Magellan 7.2. was used to collect the ELISA data (absorbance at 405nm and 620nm); MassLinks and MassLynx 4.1 were used to control the MS.
Data analysis	BIAevaluation 4.1 Software and Biacore T200 Evaluation Software 3.0 to evaluate and derived binding kinetics and affinities from SPR senorgrams; MassLinks and Max-Ent Software to process MS data; MaxQuant Software 1.6.1.0 to evaluate MS/MS data and identify peptides; DynamX 3.0 to determine peptide deuterium incorporation; Clustal Omega to align multiple amino acid sequences; PyMol Software 1.3 to visualize and inspect crystal structures; GraphPad Prism 8.0.0 to plot data and perform statistical analysis.

For manuscripts utilizing custom algorithms or software that are central to the research but not yet described in published literature, software must be made available to editors/reviewers. We strongly encourage code deposition in a community repository (e.g. GitHub). See the Nature Research guidelines for submitting code & software for further information.

Data

Policy information about availability of data

All manuscripts must include a data availability statement. This statement should provide the following information, where applicable:

- Accession codes, unique identifiers, or web links for publicly available datasets
- A list of figures that have associated raw data
- A description of any restrictions on data availability

Data that support the findings of this study are available from the corresponding author upon reasonable request.

Field-specific reporting

Please select the one below that is the best fit for your research. If you are not sure, read the appropriate sections before making your selection.

☒ Life sciences ☐ Behavioural & social sciences ☐ Ecological, evolutionary & environmental sciences

For a reference copy of the document with all sections, see nature.com/documents/nr-reporting-summary-flat.pdf

Life sciences study design

All studies must disclose on these points even when the disclosure is negative.

Sample size	Animal studies: The number of mice per group was chosen based on sample size used in published articles reporting on half-life studies in the human FcRn transgenic mice.
Data exclusions	No data were excluded from the analyses.
Replication	Half-life measurements of albumin variants were performed once in mice lacking endogenous albumin (Tg32-Alb-/- mice) and once in mice expressing albumin (Tg32 mice).
Randomization	Mice of different size/weight were distributed randomly between the different experimental groups.
Blinding	The investigators who performed the injections were blinded to what variant he/she was injecting (variants were censored and named variant 1 and variant 2).

Reporting for specific materials, systems and methods

We require information from authors about some types of materials, experimental systems and methods used in many studies. Here, indicate whether each material, system or method listed is relevant to your study. If you are not sure if a list item applies to your research, read the appropriate section before selecting a response.

Materials & experimental systems

n/a	Involved in the study
☐	☒ Antibodies
☐	☒ Eukaryotic cell lines
☒	☐ Palaeontology
☐	☒ Animals and other organisms
☐	☒ Human research participants
☒	☐ Clinical data

Methods

n/a	Involved in the study
☒	☐ ChIP-seq
☒	☐ Flow cytometry
☒	☐ MRI-based neuroimaging

Antibodies

Antibodies used	Polyclonal anti-albumin goat antibody, Sigma, Cat. nr: A1151-1VL, Lot. nr: SLBV5010<br>Monoclonal anti-HSA mouse antibody, Abcam, Cat. nr: ab10241, 15C7, Lot. nr: GR3197685-5<br>HRP-conjugated monoclonal anti-HSA mouse antibody, Abcam, Cat. nr: ab24458, Lot. nr: GR248431-1<br>ALP-conjugated polyclonal anti-HSA goat antibody, Bethyl, Cat. nr: A80-229AP
Validation	The polyclonal anti-albumin goat antibody from Sigma was validated by immunoelectrophoresis against human albumin and have been used in the same ELISA set-up in published papers, including PMID: 29434196.<br>The unconjugated and HRP-conjugated monoclonal anti-HSA mouse antibodies from Abcam have been validated for use in several applications, including ELISA. The ALP-conjugated polyclonal anti-HSA goat antibody from Bethyl was validated for use in several applications, including ELISA, and have been used in the same ELISA set-up in published papers, including PMID: 29434196.

Eukaryotic cell lines

Policy information about cell lines

Cell line source(s)	HEK293E cell line from ATCC (VA, USA), Cat nr: CRL-10852; High Five Insect cells from Thermo Fisher Scientific (MA, USA), Cat nr: B85502.<br>HMEC-1 stably expressing HA-hFcRn-EGFP was a gift from Dr. Wayne I Lencer at Boston Children's Hospital, Harvard Medical School and Harvard Digestive Disease Center.
Authentication	The cell morphology is monitored using a light microscope every time the cells are handled.

Mycoplasma contamination	All cell lines tested negative for mycoplasma contamination.
Commonly misidentified lines (See ICLAC register)	No commonly misidentified cell lines were used.

Animals and other organisms

Policy information about studies involving animals; ARRIVE guidelines recommended for reporting animal research

Laboratory animals	Mouse, Human FcRn transgenic C57BL/6 (Tg32), Male, 7-8 weeks;<br>Mouse, Human FcRn transgenic and albumin knock out C57BL/6 (Tg32-Alb-/-), Male, 11-16 weeks
Wild animals	The study did not involve wild animals.
Field-collected samples	The study did not involve samples collected from the field.
Ethics oversight	The Animal Care and Use Committee at The Jackson Laboratory approved the study protocol.

Note that full information on the approval of the study protocol must also be provided in the manuscript.

Human research participants

Policy information about studies involving human research participants

Population characteristics	The patient with acute pancreatitis was a female.
Recruitment	The patient with acute pancreatitis was identified through high serum amylase and lipase results.
Ethics oversight	The study was carried out in accordance with the Declaration of Helsinki principles and the Canterbury Health Laboratories guidelines on clinical samples.

Note that full information on the approval of the study protocol must also be provided in the manuscript.
